# Patient organizations involvement in healthcare: a rapid review and conceptual framework

**DOI:** 10.1186/s12992-026-01205-z

**Published:** 2026-03-26

**Authors:** Arianna Gentilini, Iva Parvanova, Samra Ghazi, Kristy M. Scarfone, Jillian Kohler

**Affiliations:** 1 Centre for Health Economics & Policy Innovation, Department of Economics & Public Policy, Imperial College London, London, UK; 2 Department of Political Science, LUISS Guido Carlo, Rome, Italy; 3 Leslie Dan Faculty of Pharmacy, University of Toronto, Toronto, ON M5T 3M7 Canada

**Keywords:** Patient organizations, Conflicts of interest, Health technology assessment, Healthcare

## Abstract

**Background:**

Patient organizations have become increasingly influential in the healthcare sector, offering unique insights into unmet health needs, disease impact, and patients’ quality of life. These groups have evolved from advocates for access to treatment, as seen during the HIV/AIDS crisis, to active participants in the research and policy-making processes. Despite growing recognition of their critical role, a comprehensive understanding of their interactions with various healthcare actors remains limited.

**Main body:**

This rapid review aims to map the landscape of patient organization involvement in healthcare, particularly in high-income settings. We conducted a systematic search of MEDLINE, focusing on literature published between 2000 and 2024, and identified 61 relevant articles. The analysis revealed that patient organizations interact with a range of actors, including pharmaceutical companies, healthcare professionals, payers, health technology assessment bodies, and regulatory agencies. Key themes were identified around conflicts of interest, especially with regards to the pharmaceutical industry, where concerns about transparency and independence were prevalent. The review also highlighted the vital role patient organizations play in research and development, regulatory approval processes, and reimbursement decisions. The conceptual framework developed from this review outlines these interactions across the pharmaceutical lifecycle, emphasizing the varied and significant contributions of patient organizations.

**Conclusion:**

This review underscores the need for more transparency and meaningful engagement of patient organizations in healthcare decision-making. While their involvement has been primarily studied in the context of pharmaceutical industry relations, further research is needed to explore their interactions with other relevant actors. Addressing funding challenges and expanding research beyond well-studied regions are crucial for fully understanding and optimizing the role of patient organizations in healthcare.

**Clinical trial number:**

Not applicable.

**Supplementary Information:**

The online version contains supplementary material available at 10.1186/s12992-026-01205-z.

## Introduction

Patient organizations have an increasingly important role in the healthcare sector. With members who often have lived experience of what it is like to manage various health conditions, patient organizations can advocate for healthcare systems to address unmet needs and to understand the impact of these conditions on patients’ quality of life [1].

Patient-driven advocacy first gained momentum during the HIV/AIDS crisis when grassroots organizations mobilised to influence policymakers and secure access to life-saving antiretroviral medications [2, 3]. This movement catalysed a broader recognition of the unique perspectives that patients can bring to healthcare. Building on this momentum, patient organizations now advocate across a wide range of health conditions and are active in many countries [4–6]. While their activities can move healthcare away from a paternalistic approach and towards one that is patient-centred, their relationship with other actors, including the pharmaceutical industry, must be better understood. In this review, we use the term patient organizations to refer to non-profit organisations that represent the interests of patients and/or caregivers and whose membership or governance includes people affected by a specific condition or set of conditions, consistent with common definitions in the reviewed literature [7]. We focus on organisational involvement through patient organizations rather than individual patient involvement activities (e.g., consultations, training initiatives, or engagement programmes that may be organised by public institutions or industry). The term healthcare actors is used, on the other hand, to describe institutions and professional groups that shape the development, regulation, assessment, and use of health technologies and services (e.g., manufacturers, regulators, payers bodies, governments, and healthcare professionals).

During the research and development phases, patient organizations can participate in shaping research agendas and developing study designs that incorporate patient-relevant outcomes. This can help to ensure that clinical trials consider quality-of-life measures and symptom management strategies that are meaningful to patients [5, 8–10]. Patient organizations also contribute their perspectives in regulatory and health technology assessment (HTA) processes, where they provide insights on their lived experience with the disease and help decision-makers understand how new treatments can improve (or not) their quality of life [4, 11–14]. A notable subset of the literature focuses on rare disease patient organizations, particularly in relation to research and development and regulatory engagement, reflecting both unmet need and the practical challenges of trial design and recruitment in small populations [5, 9, 14–17]. Also, as civil society actors, patient organizations often depend on external funding from a variety of private and public institutions, including the pharmaceutical industry. While funding is necessary to ensure that patient organizations can fulfil the variety of functions listed above, it raises potential concerns around conflicts of interest and credibility of their demands [7, 18–21].

Despite patient organizations’ engagement in healthcare research and policy gaining attention in the literature [9, 22–24], research-based syntheses that compare and map their involvement with different healthcare actors across decision-making settings remain limited. The objective of this review is thus twofold. First, it aims to characterise the peer-reviewed literature on patient organizations by mapping (a) which healthcare actors are examined, (b) which forms of involvement are described, and (c) the key thematic issues addressed in research. Second, it develops a conceptual framework that synthesizes how patient organizations engage with healthcare actors across key stages of the pharmaceutical lifecycle, with a focus on Europe and North America. These regions were chosen because patient-organisation engagement is comparatively more formalised and well-documented within regulatory and HTA decision-making [25]. This allows us to synthesize evidence from settings where patient organization engagement is more frequently formalized in regulatory and HTA processes. While insights may be transferable to other contexts, differences across health systems should be taken into account, providing insights that can be adapted and applied to other contexts. In addition to mapping actors and forms of involvement, we examine how the literature addresses cross-cutting issues such as conflicts of interest, transparency, and the governance arrangements that may impact the independence and credibility of patient-organization participation. Finally, we identify gaps in the existing literature and areas for further research.

The rest of the review is structured as follows: The methods and results sections discuss the search strategy, study selection, data analysis, and the findings of the rapid review. The *Conceptual Framework* section examines which institutional actors patient organizations engage with and the activities they jointly undertake. Finally, the discussion and conclusions sections present policy implications, highlights limitations, and provides concluding remarks.

## Methods

Rapid scoping literature reviews allow researchers to summarise a select amount of high-quality evidence in a timely and cost-effective manner. They are a type of knowledge synthesis in which the steps of the systematic review are adjusted to the knowledge user’s needs, producing relevant evidence in a shorter timeframe [26]. Given the scope of this review, which aims to summarize evidence on patient organizations, this approach was considered as appropriate.

### Search strategy

The protocol for this rapid review was guided by the Preferred Reporting Items for Systematic Reviews and Meta-Analyses (PRISMA) where applicable. The protocol was developed a priori by the team but was not pre-registered, consistent with many rapid scoping review approaches. We searched MEDLINE (via Ovid) for patient organizations-related articles published between January 2000 to December 2024. This database was selected because it provides broad coverage of biomedical and health policy journals relevant to this topic. The full list of search terms can be found in the Appendix. We used a combination of terms relating to patient organizations (e.g., “patient groups”, “patient organizations”, “patient advocacy organizations”) and relevant actors, including the pharmaceutical industry, physicians, regulators, payers, and policymakers in general (e.g., “drug industry”, “health technology assessment”, “FDA”). Results were restricted to publications in English only. Search terms were structured to encompass both American and British spelling by using an asterisk to account for variations. Additional articles were identified by hand searching using citations and reference lists of the included articles.

Only peer-reviewed empirical studies using primary or secondary data (e.g., policy analyses, literature reviews, interviews) were included in the analysis, while commentaries, abstracts, journal news, and other non-peer-reviewed records were excluded. Because the focus of this review is organizational engagement, we excluded studies primarily focused on individual-level clinical experiences or patient-reported outcomes in relation to treatment or disease management, unless they explicitly examined interactions between patient organizations and healthcare actors. We also required that such engagement be a primary object of analysis. Consequently, we may have excluded papers in which patient organizations were involved through co-authorship, acknowledgement of advisory roles, or participation as representatives within broader evaluations of patient and public. For example, studies co-authored by patient representatives and clinicians that primarily described a specific medical intervention did not meet our inclusion criteria. Table 1 summarizes the inclusion and exclusion criteria. The full search strategy can be found in the Appendix.

### Study selection

Identified publications underwent a two-stage exclusion-inclusion process through Rayyan, an online systematic review software program [27]. Titles and abstracts were independently screened by three authors (AG, SG, KS), with disagreements resolved through discussion or consultation with the lead author (AG). Articles that remained inconclusive after title and abstract screening proceeded to the full-text screening stage. Records that passed the first screening underwent reassessment by one author (AG) based on their full text.

**Table 1:** Review inclusion and exclusion criteria

Category	Inclusion Criteria	Exclusion Criteria
Language of publication	English	Published in other than English
Year of publication	January 2000 – December 2024	Published before 2000
Geographic scope	Europe and North America, including literature with an international or comparative focus	Articles exclusively outside of Europe or North America
Study type	Peer-reviewed original articles, analyses, literature reviews, meta-analyses	Non peer-reviewed literature (e.g., news, reports), commentaries, editorials, conference abstracts (i.e., no full-text available), clinical studies
Outcomes	Articles included must discuss patient organizations’ activities and their relationship to actors in the healthcare sector (e.g., pharmaceutical industry, regulators, payers, and physicians)	Articles discussing patient-reported outcomes, not discussing ties between patient organizations and key healthcare actors, and/or discussing patient organizations or other stakeholders alone
Methodology	Observational studies, surveys, interviews, and more	No methodology constraint was applied

### Data analysis and synthesis

Records that passed both title-abstract and full-text screening steps were included for data extraction using a form designed a priori based on research objectives in Microsoft Excel, initially informed deductively by the lead author and iteratively updated throughout the review process. Key information extracted included the record’s citation details, author affiliations, geographic scope, year of publication, study design, and outcomes. Where reported, we extracted information on study funding. However, we did not conduct a systematic analysis of author-level conflicts of interest across included publications. Additionally, terms used to refer to patient organizations, and their relative definitions (when available) were extracted. Themes identified, such as involvement of patient organizations in drug reimbursement decisions, and information on the actors with whom patient organizations interacted (e.g., regulators) were also coded from the included articles. We used an iterative coding approach to identify themes inductively from included records. An initial extraction framework was developed deductively based on the research questions and then refined during extraction as new concepts were identified. The final themes identified reflect the review team’s synthesis of the literature. Data analysis was conducted using a narrative synthesis approach. As this study is a rapid scoping review aimed at mapping available evidence and identifying key themes and gaps, rather than assessing effects, a formal risk-of-bias analysis was not undertaken, consistent with scoping review methodology [28]. Patients or patient organizations were not directly involved in the design, conduct, or interpretation of this rapid review.

## Results

**Fig. 1:**
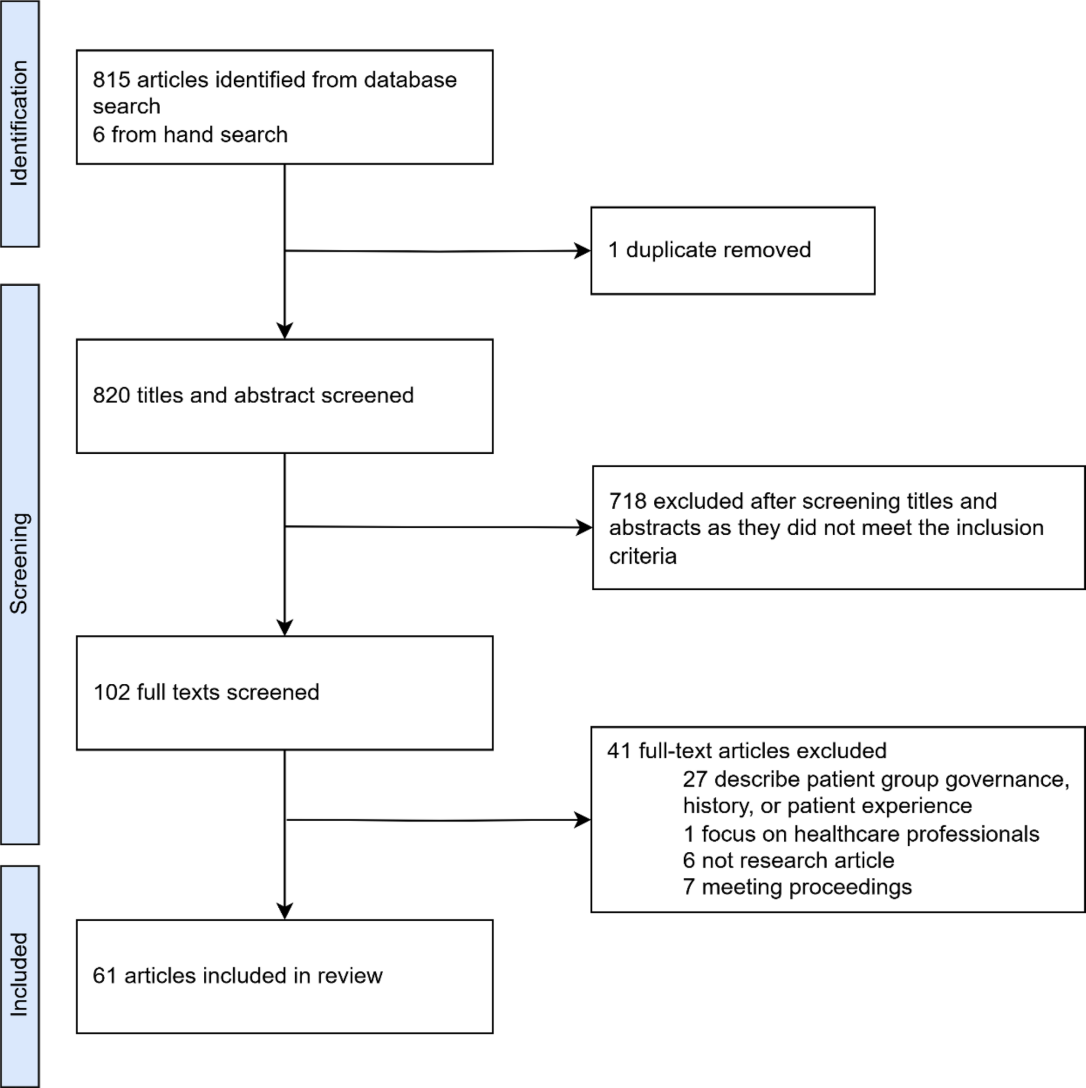
PRISMA flow diagram

The search included articles published between January 2000 to December 2024. A total of 815 articles were identified through MEDLINE, with an additional six articles included via citation screening. Full-text articles were retrieved and reviewed for 102 records. At full-text review, an additional 41 articles were excluded as they did not meet the inclusion criteria. Thus, 61 were included for evidence synthesis (Fig. 1). The list of included articles along with their key findings is provided in the Appendix.

### Study characteristics

Despite the study period spanning 2000–2024, all included articles were published post-2006, with a substantial portion (*n* = 39; 64%) of publications being published from 2018 to 2024. Most of the included articles focused on the United States (US) (*n* = 15; 25%), followed by studies with an international or multi-country scope (*n* = 11; 18%), the United Kingdom (UK) (*n* = 9; 15%), Canada (*n* = 7; 11%), Europe (*n* = 6; 10%), and the Netherlands (*n* = 4; 6.6%). Other countries were represented, although to a lesser extent, including Sweden, Denmark, and Finland (each *n* = 2; 3%), as well as France, Italy, Australia, and Poland (each *n* = 1; 2%). Many of the studies were observational analyses of secondary data (*n* = 23; 38%), followed by surveys (*n* = 12; 20%) and interviews (*n* = 9; 15%). Nine articles specifically focused on rare disease patient organizations and their role in research and development (R&D).

### Terminology and definitions used to describe patient organizations

Patient organizations were described using various terminologies in the included articles. They were most frequently described interchangeably as “patient organisation/organization” (*n* = 27; 44%), “patient advocacy organisations/organizations” (*n* = 14; 23%), and “patient group” (*n* = 11; 18%). The term “advocacy organisations/organizations” was predominantly used in the North American context, specifically in Canada and the United States, while “patient organisation/organization” was mostly used in European studies. The term “patient organizations” was used in most studies and is therefore maintained throughout this article.

While not all articles provided a definition of patient organizations, there were several key features that defined them. First, patient organizations are defined as non-profit organizations, highlighting their charitable remit [7, 18]. Second, to be defined as such, these groups ought to represent the interests and needs of patients and their families and/or their caregivers [29, 30]. Finally, their membership is defined as mainly composed by patients, families, or caregivers affected by specific medical conditions [31, 32].

These articles also noted some of the common activities and objectives of patient organizations, including raising awareness about specific medical conditions, engaging in advocacy to influence healthcare policies and practices, representing patient interests with healthcare professionals, institutions, and in the media, and supporting patients and their families through education, counselling, and information dissemination. Table 2 below illustrates how patient organizations terminology is used in the reviewed literature.

**Table 2:** Terms used interchangeably to define patient organizations in the literature

Term used*	Num. articles	References
Patient organizations	27	Ball (2006); Gentilini and Parvanova (2023); Gentilini and Rana (2024); Egher, (2023); Hansen (2020); Harmark (2020); Hemminki (2010); Lexchin (2022); Makowska, (2024); Mandeville (2019); Mankell (2021); Marvis (2012); Matos (2019); Minh, (2024); Moreira (2015); Mulinari (2020); Mulinari (2022); Nguyen (2022); Noordman, (2010); Ozieranski, (2019); Ozieranski, (2020); Ozieranski, (2022); Pashley, (2022); Polich, (2012); Rickard, (2019); Scott, (2017); Van de Bovenkamp, (2011)
Patient advocacy organizations	14	Bhat, (2023); Brems (2019); Guuidice (2022); Kang (2015) Kondamuri (2019); Li (2019); McBride Folkers (2019); McCoy (2017); Rose (2013); Rose (2015); Rose (2017); Rose (2017); Somers, (2024); Stein (2018)
Patient groups	11	Bloom (2018); Colombo (2012); Fabbri (2019); Khabsa (2020); Leto di Priolo (2012); Lexchin (2019); Lexchin (2022); Mercer (2020); Nahuis and Boon (2011); Parker (2019); Perry (2021)
Patient advocacy groups	4	Hicks (2016); Obici (2023); Patterson (2023); Rozmovits (2018)
Advocacy organizations	2	Furlong (2015); Gottlieb (2013)
Patient and consumer groups	2	Fattal (2008); Gesbert (2021)
Patient interest groups	1	Hughes (2013)

* Please note that we identified unique patient organization definitions, disregarding differences in UK/US spelling (e.g., “patient organisation” vs. “patient organization”) and singular/plural forms (e.g., “patient organisation” vs. “patient organisations”)

### Engagement with healthcare actors and key themes identified

**Fig. 2:**
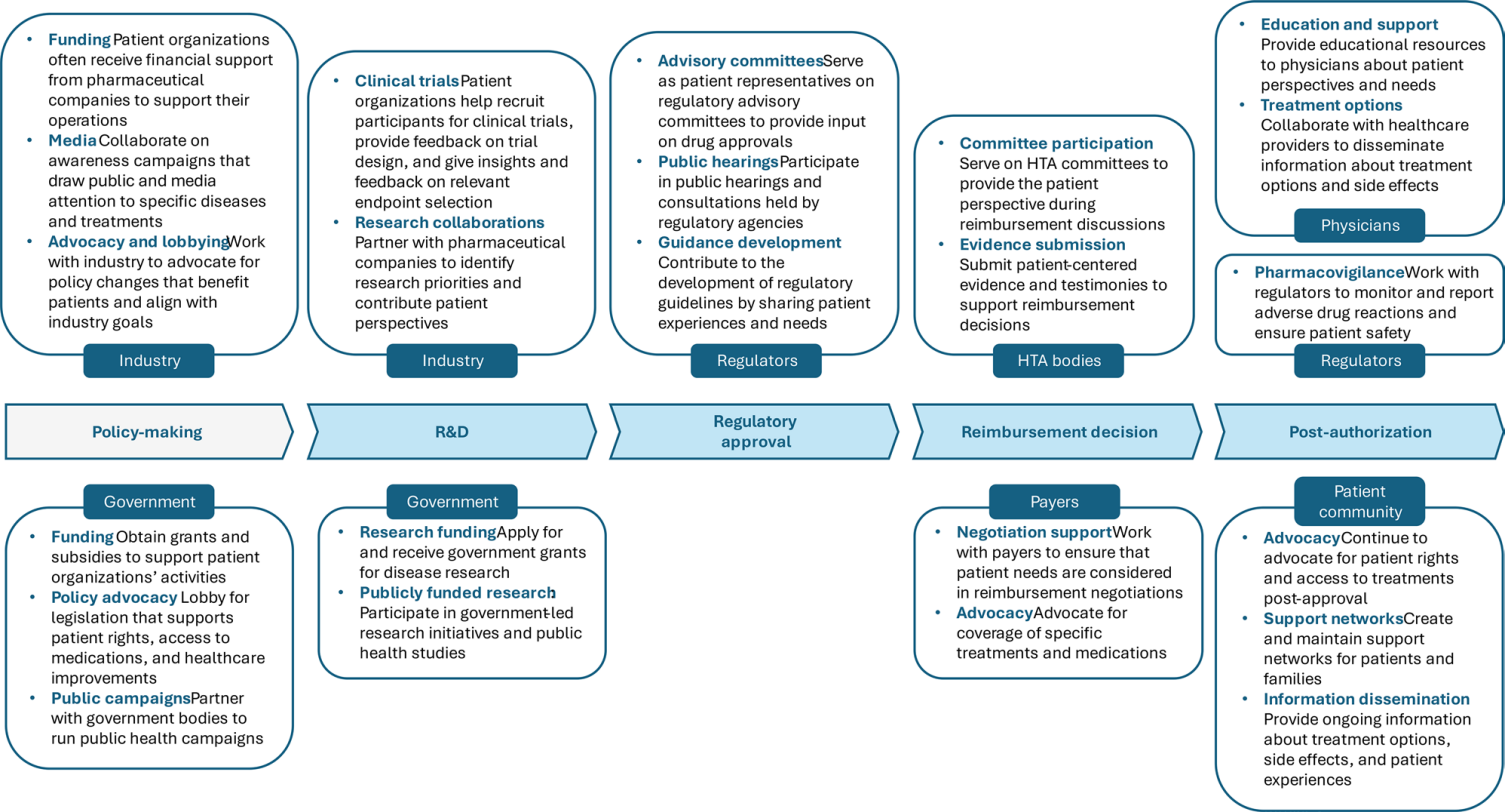
Conceptual framework of patient organizations’ engagement with healthcare actors across the pharmaceutical lifecycle

The articles included in the review discussed the engagement of patient organizations with several actors. While patient organizations often interact with multiple actors simultaneously or across different processes, the following sections are structured by institutional actors, which are therefore not mutually exclusive. In other words, one article might illustrate how patient organizations collaborate with both industry and academia to advance R&D, resulting in the article appearing under both actor categories. Table 3 summarizes the themes identified by institutional group, describes these interactions, and indicates the relative references. Figure 2 complements Table 3 by mapping these interactions across the pharmaceutical lifecycle and indicating the direction of engagement where reported. Each of the sections below illustrates the themes discussed in the literature in relation to these specific actor interactions.

**Table 3:** Summary of key themes identified by institutional actors

Actor(s)	Theme(s)	Description	References
Industry	Conflicts of interest	Pharmaceutical industry ties with patient organizations and resulting COIs, covering transparency in funding, financial relationships, and their role in promoting industry products.	Ball (2006), Bhat (2023), Colombo (2012), Del Giudice (2022), Fabbri (2019), Gentilini and Parvanova (2023), Gottlieb (2013), Hemminki (2010), Kang (2015), Khabsa (2020), Kondamuri (2019), Leto di Priolo (2012), Lexchin (2022), Lexchin (2022), Li (2019), Makowska (2024), McCoy (2017), Mulinari (2020), Mulinari (2022), Ozieranski (2019), Ozieranski (2020), Ozieranski (2022), Parker (2019), Pashley (2022), Rickard (2019), Rose (2013), Rose (2017), Rose (2017), Somers (2024), Stein (2018)
	R&D	Collaboration between industry and patient organizations in R&D activities.	Bloom (2018), Egher (2023), Hicks (2016), Nguyen (2022), Patterson (2023), Perry (2021), Polich (2012), Rose (2015), Stein (2018)
	Involvement in reimbursement decisions	Influence of industry on patient organizations’ involvement in drug reimbursement decisions.	Lexchin (2019)
Payers and HTA bodies	Involvement in reimbursement decisions	Engagement of payers and HTA bodies with patient organizations in reimbursement decision processes.	Fattal (2008), Gentilini and Rana (2024), Gesbert (2021), Hughes (2013), Mandeville (2019), Mercer (2020), Minh (2024), Moreira (2015), Rozmovits (2018), Scott (2017)
	Conflicts of interest	COIs in interactions between payers/HTA bodies and patient organizations.	Gentilini and Rana (2024), Hughes (2013), Mandeville (2019)
Governments	Funding	Financial influence of the government on patient organizations and patient organizations’ efforts to shape policy agendas and funding priorities.	Van de Bovenkamp (2011)
	Involvement in reimbursement decisions	Influence of patient organizations on public agendas which affect reimbursement decisions.	Nahuis and Boon (2011)
Regulators, public health agencies	Involvement in regulatory decision-making process	Engagement of regulatory and public health agencies with patient organizations in decision-making processes.	Mankell (2021), Furlong (2015), Hansen (2020), Marvis, (2012)
	RWE	Collection and dissemination of real-world evidence involving patient organizations.	Matos (2019)
Healthcare professionals	Medical care	Perspectives of healthcare professionals on treatment options and patient care through engagement with patient organizations.	McBride Folkers (2019), Obici (2023)
Other	RWE	Pharmacovigilance activities involving patient organizations.	Harmark (2020)
	R&D	Collaboration with academia on research and development involving patient organizations.	Polich (2012)

Abbreviations: COI, conflicts of interest; HTA, Health Technology Assessment; R&D, research and development; RWE, real-world evidence

#### Industry

Most of the identified literature discussed the relationship between patient organizations and the pharmaceutical industry (*n* = 39, 64%). A primary theme that was identified in relation to the pharmaceutical industry was conflicts of interest (COI), that was discussed in 30 papers (49%), across multiple geographical locations, including the United States (US), United Kingdom (UK), Canada, Denmark, Sweden, Finland, Italy, Poland, and broader European contexts. Overall, articles discussing COIs were broadly divided into two groups: those describing the financial payments from industry to patient organizations, and those focusing on the policies and disclosure practices in place to reduce the impact of such conflicts and lack of transparency in the relationship.

Regarding the reliance on industry funding, many articles identified pervasive but often underreported financial ties between industry and patient organizations [7, 18, 33–37]. Although most patient organizations were found not to be financially dependent on industry funding, a UK study revealed that a few organizations significantly relied on industry funding, raising concerns about potential co-optation, where strong financial reliance shape organizational agendas, advocacy priorities, or public messaging to align more closely with industry interests [7, 19]. In Denmark and Sweden, funding patterns revealed dominance by a few pharmaceutical companies [34, 38]. This raised concerns that the influence of a few dominant pharmaceutical companies could shape the activities and priorities of patient organizations, and that funding might not be distributed fairly across different groups or disease areas, creating inequalities in access to resources. Companies were also found to predominantly fund patient organizations that operated in therapeutic areas relevant to their portfolio or drug development pipeline [7]. Additionally, the food industry in the US provided substantial donations to patient organizations that focus on non-communicable diseases, raising ethical concerns and potential COIs [39].

Articles on funding transparency and COI policies identified several important issues, including the lack of central and public databases to declare payments made to patient organizations and the unclear or unenforced regulations to mitigate conflicts. A survey in Italy indicated poor transparency on websites regarding industry funding, with significant discrepancies between reported funding by pharmaceutical companies and patient organizations [40]. In Canada, articles revealed inconsistent disclosure practices by both pharmaceutical companies and patient organizations, with significant gaps in the reporting of financial relationships [29, 41]. In the US, an analysis of 47 patient organizations found that a substantial proportion had policies addressing individual (a reported 85%) and institutional (a reported 51%) COIs. However, details about what was done to restrict corporate partnerships and disclosing financial values were often lacking [42]. Another US-based study found that while most patient organizations received industry funding, few had robust COI policies in place, highlighting the need for management of COIs and improved transparency [20]. In Europe, disclosure practices varied significantly across Nordic countries, with transparency often lacking in Norway and Finland compared to Sweden and Denmark [36]. UK studies identified inadequate transparency in payment disclosures, as many payments were not clearly described, complicating efforts to manage potential COIs [37, 43]. A systematic review highlighted that financial relationships between patient organizations and the health industry are common but often not disclosed, necessitating stricter regulations to ensure transparency and independence [44]. The literature also indicates that self-regulation has proven ineffective in improving transparency in the relationship between patient organizations and the pharmaceutical industry [36].

Two US studies further explored the dynamics of these relationships. One study surveyed 289 patient organizations’ leaders, revealing that 67.3% reported receiving industry funding, with a minority receiving substantial support, raising concerns about independence. Many patient organizations acknowledged the need to improve their COI policies to maintain public trust [21]. Another study analysed tax records, annual reports, and websites of 104 US-based patient organizations, finding that 88% received financial support from industry, with many having industry executives on their governing boards, indicating a potential for significant influence [18].

A systematic review with meta-analysis found that industry funding of patient organizations is common, with prevalence estimates ranging from 20% to 83% [45]. However, transparency about this funding is often inadequate, and few patient organizations have policies which govern corporate sponsorship. Industry-funded groups are also found to support sponsors’ interests, highlighting the need for strategies to prevent biases favouring industry over public interests [45]. This finding was also echoed by an Australian study which identified varying attitudes towards pharmaceutical industry sponsorship among patient organizations, with some viewing it as a successful business partnership while others saw it as incompatible with their missions as it could skew patient organization activities towards industry interests [46].

Another important theme focused on the collaboration between patient organizations and pharmaceutical companies in the R&D process. Many articles focused on how this relationship should be established and how it can be effective and transparent. For example, a US study highlighted how guidelines for collaborations between patient organizations and biopharmaceutical companies should enable ethical, transparent, and mutually beneficial interactions [16]. Another study discussed the development of a prioritisation tool to streamline patient organization engagement in clinical trials, identifying key benefits such as increased patient recruitment, improved study design relevance, and enhanced credibility of trial outcomes, along with necessary investments like training for patient representatives and infrastructure support for continuous engagement [47]. In the EU, qualitative interviews with pharmaceutical industry representatives, regulatory authorities, and patient organization representatives identified key perceptions and barriers to effective patient involvement in drug development. The study underscored the need for trust between the actors involved, clear frameworks, and a cultural mindset change within the pharmaceutical industry. This means fostering an environment where patient input is valued and integrated into the decision-making processes, ensuring that both parties understand their roles and expectations clearly [48]. Interestingly, nine articles (15%) discussed the importance of involving rare disease patient organizations in R&D. This is primarily because rare disease patients can provide unique insights into disease mechanisms and unmet needs, which are often overlooked in broader research efforts. Furthermore, their involvement can accelerate the development of therapies by ensuring that research priorities align with patient needs and by facilitating patient recruitment for clinical trials [15, 49, 50]. A case study of the Pulmonary Fibrosis Foundation (PFF) exemplified successful collaboration between patient organizations and various actors in the R&D process [51]. PFF initiatives include facilitating partnerships between academia, biopharma, public funding agencies, and regulatory bodies, establishing research and clinical trial networks, and maintaining an Industry Advisory Council to advance drug discovery and development.

#### Payers and HTA bodies

Several articles discussed the increasingly important role that patient organizations play in HTA processes, where they contribute valuable perspectives to reimbursement and treatment decisions (*n* = 10; 16%). One study examined the perception of patient organizations submitting evidence as part of Canada’s pan-Canadian Oncology Drug Review (pCODR) [52]. While the patient organizations viewed their ability to make submissions as meaningful, they explained that they faced resource challenges, including high opportunity costs and difficulty accessing necessary literature. Furthermore, they expressed uncertainty about the impact of their submissions. Similarly, an international survey of patient organizations involved in HTA processes revealed varying levels of support from HTA agencies [53]. The survey indicated that while patient involvement is increasing, gaps remain in facilitating this involvement, providing clear roles, and ensuring effective communication between patient organizations and HTA agencies. Interviews of reviewers and payers involved in pCODR processes found that patient submissions were considered meaningful when they provided unique information not available from other sources, such as insights on clinical trade-offs and lived experiences [54]. However, submissions relying on emotional appeals or lacking transparency were seen as detracting from the process, highlighting the lack of alignment between actors regarding what constitutes meaningful patient engagement [54]. A recent UK study found that patient organizations contributing to NICE appraisals of medicines for ultra-rare diseases primarily shared insights based on lived experience, such as disease burden, access challenges, and mental health impacts. However, only about half of these inputs were explicitly reflected in final decision documents, raising uncertainty about the extent to which they influenced the decision-making process [13].

Issues of equity were raised in one of the articles, which found that it is the larger patient organizations were more likely to be successful in their advocacy efforts, specifically in their demands for reimbursement of medical products [55]. Concerns about COIs were also discussed in reimbursement processes. Mandeville and colleagues investigated the financial interests of patient organizations contributing the UK HTA body, the National Institute for Health and Care Excellence (NICE), found that 72% of these organizations had accepted funding from manufacturers of the technologies being appraised or their competitors. Moreover, NICE’s disclosure policies were found to be inadequate, with decision-making committees aware of less than a quarter of these financial interests [30].

Another UK study found that most patient organizations submitting written evidence to NICE during ultra-rare disease appraisals had financial relationships with the company whose product was under review, with inconsistent disclosure requirements across appraisals [13]. Similarly, a study in Quebec, Canada, examined the role of a patient organization called Coalition Priorité Cancer (CPC) in the reimbursement process of expensive cancer drugs [56]. The analysis indicated that the organization’s substantial industry funding raised concerns about its independence, especially in light of its role in the reversal of a decision by the Institut National D’Excellence en Santé et an Service Sociaux (INESSS), Quebec HTA body, not to recommend reimbursement for certain cancer drugs due to cost-effectiveness concerns [56]. Finally, articles investigated different modalities of involvement for patient organizations with HTA bodies. For example, in France, an online-based system was implemented for patient organizations to submit their contributions to the French HTA body, the Haute Autorité de Santé (HAS) [57]. The system was found to be effective, with 79 contributions from 44 patient organizations received in two years, covering quality-of-life aspects, access to care, and personal and family impact. Also, the online platform was considered to have the potential to address some challenges faced by patient organizations in the reimbursement process, such as time constraints and limited financial resources [57].

#### Governments, regulators and public health agencies

While most articles exploring the relationship between patient organizations and public institutions concentrated on HTA and payers, a few also examined their interactions with other public sector organizations, including governments and regulators (*n* = 7; 11.5%). The relationship between patient organizations and the government often involves funding and influence over organizational activities and policies. In Sweden, a study explored how patient organizations were represented within a decentralised healthcare system [58]. The study found that, although there were structures in place for patient organization representation, challenges arose in balancing democratic ideals with managerial practices, leading to the professionalisation of civil society organizations and ultimately creating a conflict between democratic values and effective management.

In the Netherlands, government influence on patient organizations was found to be substantial [59]. The study revealed that government funding affects the organizational structure, activities, as well as the ideology of patient organizations. While government subsidies are seen to reduce dependence on pharmaceutical industry funding, they are also discussed as a source of government influence on civil society.

Regarding the role of patient organizations in regulatory processes, the literature particularly focused on their involvement in approving new therapies for rare diseases. For example, in the US, a patient organization focused on muscular dystrophy developed a draft guidance document for industry submission to the FDA [60]. This initiative addressed the challenges of conducting clinical trials in small populations and aimed to facilitate the development of safe and effective therapies for rare diseases. Another study noted how the European Medicines Agency (EMA) has included members of patient organizations as full members with equal voting rights in the Committee for Orphan Medicinal Products (COMP), allowing patients to have a direct role in regulatory decision-making processes, particularly for orphan drugs [50].

#### Healthcare professionals

Finally, the literature discussed how patient organizations and healthcare professionals collaborate to improve patient care (*n* = 2; 3%). These collaborations often involve multidisciplinary panels and joint efforts to enhance care and treatment options. One Delphi survey involving patient organizations and healthcare professionals across 27 countries, underscored how guidance was developed for the holistic care of hereditary ATTR amyloidosis [61]. This guidance covered early diagnosis, treatment, disease monitoring, organization of care, physical and mental health maintenance, family-centered care, patient-doctor dialogue, and social support. A literature review highlighted patient organizations’ contributions in clinical research, such as documenting treatment benefits and adverse effects and evaluating new uses for off-label drugs [15]. Patient organizations were also found to provide vital information on pre-approval access to investigational treatments [62]. Their analysis of 118 patient groups’ websites revealed that a majority provided links to ClinicalTrials.gov and relevant trials.

#### Others

Finally, two articles (3%) were found discussing the relationship between patient organizations and other actors, namely academia and the patient community. Engagement with academia is particularly important in the context of R&D, where patient organizations support researchers in the early stages of medicine development to ensure that patient-relevant aspects are captured in trials and translational research [15]. Another article explored how adverse drug reactions can be communicated through patient organizations to their members via newsletter, social media channels, website, and print magazine, using a case study implementation by a Dutch thyroid patient organization. The results suggested that patient organizations hold great potential in effectively communicating adverse drug reactions to patients in a clear and easy-to-understand manner, highlighting their significant impact in disseminating important health information [63].

### Conceptual framework

Based on the results of a rapid review, we have developed a conceptual framework outlining the engagement of patient organizations with various healthcare actors throughout the pharmaceutical lifecycle. Framework development was primarily informed by the empirical patterns and themes identified in the included studies, specifically the interactions of patient organizations with key actors (i.e., industry, governments, payers, HTA bodies, regulators, public health agencies, and physicians) and the domains of engagement described. We first mapped reported forms of involvement to key domains of medicine development, including policymaking, R&D, regulatory approval, reimbursement decisions, and post-marketing authorization, and then used an interpretive synthesis to group and refine these activities into a coherent structure. The framework was iteratively refined through team discussion to ensure that the domains and actor linkages reflected the engagement described in the included literature (Fig. 2). In the policy-making domain, patient organizations often collaborate with industry for funding, media attention, and advocacy efforts. For instance, pharmaceutical companies provide financial support to patient organizations to sustain their operations and drive awareness campaigns [46, 54, 59]. These collaborations enable patient organizations to leverage media attention and advocate for policy changes that align with their goals, often overlapping with patient needs [31, 64]. However, these could also create tensions, as industry may steer advocacy priorities or public messaging in ways that align more closely with commercial objectives than with broader patient interests [7, 19, 43].

Governments also play a crucial role by offering grants and subsidies to support patient organization activities and by partnering on public health campaigns [59, 65]. In R&D, patient organizations are integral to clinical trial recruitment and the design of research initiatives. They collaborate with pharmaceutical companies and academic institutions to identify research priorities and contribute patient perspectives [15, 17, 47]. Governments provide research funding and involve patient organizations in collaborative projects to enhance public health research [59]. During the regulatory approval process, patient organizations actively participate as representatives on advisory committees and in public hearings, providing crucial patient insights into drug approvals [48, 50, 60].

They also contribute to the development of regulatory guidelines by sharing patient experiences and needs, thus influencing the decision-making processes of regulatory agencies like the FDA and EMA. In the domain of reimbursement decisions, patient organizations engage with HTA bodies and payers to advocate for the coverage of treatments. They serve on HTA committees, provide evidence, and submit testimonies that reflect patient perspectives [30, 66]. This involvement ensures that reimbursement decisions are informed by the real-world needs and experiences of patients [54]. Post-marketing, patient organizations continue to play a vital role by advocating for patient rights, providing education and support, and disseminating information about treatment options and side effects to the patient community and healthcare providers [17, 21, 67]. They collaborate with regulators in pharmacovigilance activities to monitor and report adverse drug reactions, ensuring ongoing treatment safety and efficacy [63, 68].

## Discussion

Studies about various aspects of the relationship between patient organizations and the pharmaceutical industry make up the majority of the identified literature. Most articles focus on the ethical implications of these groups receiving funding from industry, describing financial dependency and whether it affects their behaviour [16, 18–21, 31, 35, 36, 39, 40, 42, 44–46, 67, 69–74]. One study, for example, discussed how some patient organizations that were funded by a pharmaceutical company manufacturing a blockbuster biologic drug came out strongly opposed to switching to biosimilars, citing worries about their safety which were not sustained by regulators and health professionals [38]. Other studies also explained how transparency in these financial relationships is necessary to increase trust in patient organization activities, indicating that self-regulation might be inadequate for this purpose and that a mandatory unique payment reporting system might be warranted [36]. State-level regulations have been implemented by some European countries to address the need for transparency [40, 45, 75].

One example is the Bertrand Act, adopted in 2011 in France, which mandate companies to report payments to a number of actors, such as healthcare professionals and patient organizations [76]. Despite this being a prevalent theme in the literature, some gaps remain unaddressed. For example, there is a lack of studies investigating what proportion of patient organization’s overall budget comes from pharmaceutical funding, and whether these funds have replaced other sources, such as government grants, or not. Some studies suggest that declining public funding over recent decades has pushed patient organizations to actively seek industry support, effectively leaving them without alternative sources of financial sustainability [59].

Patient organizations and the industry were also found to collaborate on R&D, particularly in the context of rare diseases. As these conditions affect a small number of people and innovation is challenging due to limited research, working together with patient organizations is deemed important for successful recruitment strategies, identifying relevant study endpoints, and understanding patient preferences [15, 50]. However, the existing literature mostly discusses case studies where patient involvement has been important or theoretically examines why patient organizations’ engagement would be relevant in advancing research. Only one study assessed the empirical role of patient organizations’ involvement in R&D in advancing innovation, however this focused on rare diseases within a European context, which may limit the generalizability of its findings.

Several articles discussed the involvement of patient groups in both reimbursement and regulatory processes. For instance, patient organizations can participate in HTA committees, providing testimonies about their lived experiences [54]. However, the overall impact of their contributions remains largely unclear, highlighting the lack of evidence on how patient testimonies on lived experience inform decision-making. This suggests a need for better alignment of actor expectations and enhanced support for the technical capacity of patient organizations.

While there is widespread agreement that patient perspectives should be included in reimbursement decisions, meaningful inclusion often requires time and support to navigate the procedural and technical requirements of HTA and reimbursement processes (e.g., evidentiary standards, submission timelines, and interpreting clinical and economic evidence), underscoring the importance of institutional support structures. A few articles have highlighted the ethical challenges of patient involvement in HTA due to industry funding, with donors potentially influencing group activities to align with industry interests [56, 65]. This raises governance concerns if training content, priorities, or participation pathways are shaped by commercial interests.

Additionally, patient involvement was found to be limited to countries where HTA systems are well-established, such as the UK, France, and Canada [11, 53, 64].

Patients’ representatives are also involved in regulatory decisions. For example, they sit in the European Medicines Agency Committee for Orphan Medicinal Products, where they are permanent and full members with voting rights [50]. The literature also explores how patient organizations engage with other actors, such as governments, healthcare professionals, academia and patients. Doctors and physicians engage with patient organizations primarily in discussions regarding treatment options and how to better provide for the patient community [61, 62]. Patient organizations were also found to work closely with academia on medical research and can play an important role in translating scientific and medical information into a lay-friendly format for patients to use [9, 51, 63]. However, the increasing involvement of patient organizations in clinical trial design appears to contrast with a trend toward the use of surrogate endpoints in trials, which do not reliably predict longer patient survival or improved quality of life [77, 78]. This apparent contradiction suggests that while patient organizations are more engaged in the R&D process, their influence may be limited to peripheral aspects such as recruitment and dissemination, rather than shaping core trial parameters like primary endpoints.

This study is not without limitations. First, it employed a rapid scoping review approach, which is less rigorous than a systematic review. As a result, some relevant articles may have been missed, particularly those not indexed in MEDLINE or not captured through reference screening. However, this is unlikely to have significantly affected the results, as MEDLINE is one of the largest and most comprehensive medical literature databases. Also, because we focused on peer-reviewed research literature, we may have missed relevant resources in the grey literature (e.g., reports by patient organizations, regulators, or reimbursement bodies). Second, the focus was on articles solely covering interactions between patient organizations and other actors, hence not accounting for evidence discussing, for example, the activities carried out by these organizations. Finally, limiting the review to English-language articles may have excluded some regional perspectives, but much of the relevant literature in the focus regions (Europe and North America) is published in English, reducing the risk of major gaps.

This study advances our understanding of the existing literature on patient organizations and their involvement with healthcare actors and formalises this engagement in a comprehensive conceptual framework, whilst being intentionally simplified and not fully capturing intersections between actors across stages or differentiate patient organizations by funding models. In particular, the framework does not explicitly model how commercial influence may cut across multiple stages and actor relationships (e.g., industry links shaping advocacy and media strategies, evidentiary submissions in HTA, and participation in regulatory consultations) nor does it differentiate between patient organizations with and without industry relationships. Future research should test and extend the framework by mapping these cross-cutting linkages and stratifying engagement patterns by funding structure and conflicts of interest. The results underscore a need to improve transparency in funding relationships and highlight the importance of meaningful patient organization involvement in various aspects of healthcare decision-making. While we extracted study funding information where reported, we did not conduct a systematic analysis of author-level conflicts of interest across included publications, which could be explored in future research to examine how funding and authorship-related COI in this literature may shape study questions, methods, and reported conclusions. Future research should focus on ways to address the existing funding dilemma for patient organizations, which are underfunded by the public sector but whose reliance on industry funding might compromise their independence and trustworthiness. One option could be to set up a third-party public body to which pharmaceutical companies would pay a set fee, which would then be allocated to patient organizations based on need. Such a process would help avoid competition between equally deserving patients and disentangle financial support from private commercial interests. Furthermore, research beyond geographical areas that have received the most attention, such as the UK and Canada, is needed to understand the full range of patient organization activities and the scope of their engagement. Finally, future work should systematically synthesise evidence on the diverse activities patient organizations carry out across the phases of the R&D spectrum outlined in the conceptual framework to provide a better understanding of scope of their engagement. Beyond industry-related COI, the literature provides comparatively less clarity on patient organizations’ roles in government policy agendas and reimbursement institutions, including how input is solicited, incorporated, and weighted in decisions across health systems. Addressing these gaps would support a more balanced evidence base on patient organization involvement.

## Conclusions

Patient organizations engage with a diverse range of healthcare actors, contributing significantly across various aspects, from R&D to reimbursement decisions and medical care. This review was used to populate conceptual framework that outlines the activities of patient organizations throughout the pharmaceutical lifecycle and beyond. Most of the existing literature has concentrated on the relationship between patient organizations and the pharmaceutical industry, particularly focusing on COI and transparency issues. There is a need for future research to explore patient engagement with other actors, such as governments, and HTA bodies, to gain a more comprehensive understanding of their roles and impacts.

## Supplementary Information

Below is the link to the electronic supplementary material.

Supplementary Material 1

## Data Availability

The datasets generated and/or analyzed during the current study are available from the corresponding author on reasonable request. All papers included in the literature review are publicly available.
