# Supplementary material for: Patient organizations involvement in healthcare: a rapid review and conceptual framework
Source: Global Health. 2026 Mar 26;22:43. doi: 10.1186/s12992-026-01205-z (PMC13141336; doi:10.1186/s12992-026-01205-z)
Supplement: Supplementary file 1 — Supplementary Material 1 [file 12992_2026_1205_MOESM1_ESM.docx]

### **Patient organizations involvement in healthcare: A rapid review and conceptual framework**

### **Appendix**

#### **Search terms and results using MEDLINE (via Ovid) database as of October 4, 2023**

| **#** | **Searches** | **Results** |
| --- | --- | --- |
| **1** | (patient$ adj3 (group$ or organi?ation$ or advoca$ or voice$ or represent$)).ti. | 8344 |
| **2** | (patient-advoca$ or patient-representat$ or patient-group$ or patient-organi?ation$).ti. | 1796 |
| **3** | 1 or 2 | 8344 |
| **4** | ((pharmaceutical$ or biopharma$ or biotechnologic$) adj3 (compan$ or industr$ or corporation$)).ti,ab. | 33379 |
| **5** | (drug indust$ or policy maker$ or policymaker$ or policy-maker$ or regulator$ or payer$ or reimbursement$ or european medicine agency or EMA or "food and drug administration" or FDA or "Agency for health care policy and research" or AHCPR or "agency for healthcare research and quality" or AHRQ or council on health care technology or CHCT or national center for health care technology or NCHCT or office of health technology assessment or OHTA or office of technology assessment or OTA or therapeutic products directorate or TPD or "Canadian Agency for Drugs and Technology in Health" or CADTH or Canadian health technology assessment collaborative or institute of health economics or Conseil d'Evaluation des Technologies de la Sante du Quebec or CETS or Canadian Coordinating Office for Health Technology Assessment or CCOHTA or British Columbia Office of Health Technology Assessment or BCOHTA or Health Technology Assessment Unit of the Alberta Heritage Foundation for Medical Research or AHFMR or Calgary Health Technology Implementation Unit or CaHTIU or "Centers for Medicare and Medicaid Services" or CMS or physician$ or doctor$ or general practitioner$ or healthcare professional$ or medical professional$ or health technology assessment or HTA).ti,ab. | 1596627 |
| **6** | (drug adj3 (development or approval or discovery or evaluation)).ti,ab. | 110341 |
| **7** | Physicians/ | 103038 |
| **8** | Reimbursement Mechanisms/ or Insurance, Pharmaceutical Services/ or Insurance, Health, Reimbursement/ | 27205 |
| **9** | Drug Industry/ | 35040 |
| **10** | drug development/ or drug approval/ or drug discovery/ or drug evaluation/ | 97279 |
| **11** | 4 or 5 or 6 or 7 or 8 or 9 or 10 | 1854076 |
| **12** | 3 and 11 | 931 |
| **13** | limit 12 to (english language and yr="2000 - 2024") | 815 |

#### **Summary of included studies**

| **Author, Year** | **Geographic scope** | **Study design** | **Patient group term used** | **Study aim** | **Results** | **Actor(s)** | **Theme(s)** | **Rare*** |
| --- | --- | --- | --- | --- | --- | --- | --- | --- |
| Ball, (2006) | US, UK, Australia, Canada, South Africa | Survey | Patient organizations | To examine disclosure of financial support, sponsorship, and advertising by companies on patient organization websites. | Patient organisation websites clearly stated their identity and goals but lacked transparency on funding sources. Few had COI policies, and while product advertising was rare, corporate logos and links were common. | Industry | COI | N |
| Bhat, (2023) | US | Descriptive analysis of secondary data | Patient advocacy organizations | To develop guidelines for effective collaborations between patient advocacy organisations and the biopharmaceutical industry. | Guidelines address patient advocacy organisations' interactions with biopharmaceutical companies, covering engagement, privacy, financial contributions, and clinical trial communication, with ethical recommendations for leaders. | Industry | COI | N |
| Bloom, (2018) | US | Interviews | Patient groups | To identify key factors for effective patient group engagement in clinical research. | The multistakeholder group identified the lack of well-defined guidelines as a major barrier to sponsor engagement with patient groups. This gap complicates engagement activities and competes with study priorities. | Industry | R&D | N |
| Brems, (2019) | US | Content analysis | Patient advocacy organizations | To analyse the content of PAO COI policies in the National Health Council. | Among 47 organisations, 51% had policies addressing institutional COIs. Few restricted corporate partnerships or specified financial disclosure processes. Most lacked ongoing review responsibilities. | Medical community | Treatment options | N |
| Colombo, (2012) | Italy | Survey | Patient groups | To assess the transparency of a large group of Italian patient and consumer groups and a group of pharmaceutical companies. | Among 17 pharmaceutical companies, 13 disclosed funding 341 groups. Disclosure varied widely on websites, with few reporting funding amounts. Industry logos and advertisements were common. Sponsorships from companies often did not match group disclosures, indicating inconsistencies. | Industry | COI | N |
| Egher,  (2023) | Europe | Thematic analysis | Patient organisations | To develop guidelines for effective collaborations between patient advocacy organisations and the biopharmaceutical industry. | Guidelines address patient advocacy organisations' interactions with biopharmaceutical companies, covering engagement, privacy, financial contributions, and clinical trial communication, with ethical recommendations for leaders. | Industry, regulators, academia, multi stakeholder groups (EUPATI) | R&D | N |
| Fabbri, (2019) | International | Systematic literature review and meta-analysis | Patient groups | To investigate pharmaceutical or medical device industry funding of patient groups. | Out of 26 studies, 15 estimated industry funding prevalence, ranging from 20% to 83%. Only 27% of patient organisations disclosed industry funding on their websites. Disclosure rates varied (0%-91%) due to different government requirements. Organisational policies on corporate sponsorship ranged from 2% to 64%. Industry-funded groups generally supported their sponsors’ interests. | Industry | COI | N |
| Fattal, (2008) | Canada | Interviews | Patient and consumer groups | To understand how and why patient and consumer organizations use HTA findings and what factors influence their communication. | HTA findings are used instrumentally, conceptually, and symbolically by patient and consumer organisations. Their mission and knowledge base influence usage, potentially widening debates on controversial health technologies. | Payers and HTA bodies | Involvement in reimbursement decisions | N |
| Furlong, (2015) | US | Case-study | Advocacy organisations | To develop and review therapies for Duchenne through a patient advocacy-initiated guidance involving a broad coalition. | PPMD created draft guidance for the FDA with over 80 stakeholders, aiming to expedite new therapy development while ensuring safety and efficacy | Regulators, public health agencies | Involvement in the regulatory decision-making process | Y |
| Gentilini and Parvanova,  (2023) | UK | Descriptive analysis of secondary data | Patient organisations | To assess the relationship between UK-based patient organisation funding and companies' commercial interests in rare and non-rare diseases in 2020 | Pharmaceutical companies made 1,422 payments to 341 patient organisations, with 90% of the funds going to groups aligned with the companies' drug portfolios and R&D. Despite rare diseases affecting under 5% of the UK population, over 20% of the payments went to rare disease groups, which depended on fewer companies for funding (p=0.0031). | Industry | COI | Y |
| Gentilini and Rana, (2024) | UK | Descriptive analysis of secondary data | Patient organisations | To characterise patient organisation contributions to HTA for ultra-rare diseases in England and Wales and evaluate how they are considered in NICE recommendations | Patient submissions mainly focused on disease aspects (54%). Patient raised concerns on access challenges, caregiver burden, and mental health. Most patient themes overlapped with manufacturer (82%) and doctor (45%) submissions, with novel insights mainly concerning access and mental health. Patient organisations often reported financial ties to manufacturers (£5,000–£74,113). About half of patient inputs were explicitly reflected in NICE final decisions. | Payers and HTA bodies | COI, Involvement in reimbursement decisions | Y |
| Gesbert, (2021) | France | Descriptive analysis of secondary data | Patient and consumer groups | To analyse contributions of PCGs participating in HTAs via a new open, online, systematic contribution process | HAS received 79 contributions from 44 PCGs for 78 of 592 HTAs on drugs or devices over two years. 25% of drug HTAs received contributions, covering quality of life, access to care, and personal and family impact. Membership and budgets of PCGs varied widely. | Payers and HTA bodies | Involvement in reimbursement decisions | N |
| Gottlieb, (2013) | US | Case-study | Advocacy organisations | To explore how a grassroots organization responded to Merck's positioning of Gardasil. | Merck’s Gardasil marketing exemplified the patient-consumer-advocate. Despite initial efforts and campaigns, long-term public engagement was limited. Gardasil’s promotion reinforced female health responsibility and patient consumerism, aligning advocacy with consumer practices. | Industry | COI | N |
| Del Giudice, (2022) | US | Descriptive analysis of secondary data | Patient advocacy organisations | To quantify food industry donations to NCD-focused PAOs | Nine food and beverage companies donated $10.7m to NCD-focused PAOs from 2001-2018, with most donations as 'matching gifts.' Cancer-focused organizations received the most donations | Industry | COI | N |
| Hansen, (2020) | Denmark | Interviews | Patient organisations | To explore how perceptions of patient organizations, pharmaceutical companies, and regulatory agencies influence patient roles in drug development | Patient involvement is seen as improving quality of life, avoiding business failure, and speeding drug approval. Transparency, trust, and clear roles are essential. Barriers include mindset changes, lack of a common framework, limited resources, and patient responsibility concerns | Regulators, public health agencies | Involvement in regulatory decision-making process | N |
| Harmark, (2020) | Netherlands | Pilot study | Patient organisations | To explore if collaboration with patient organizations can effectively communicate adverse drug reaction insights to patients. | Social media interactions were positive, enhancing communication of adverse drug reactions and generating significant engagement, strengthening patient experiences and feedback. | Other | RWE | N |
| Hemminki, (2010) | Finland | Survey & interviews | Patient organisations | To investigate the cooperation between patient organizations and the drug industry in Finland. | Most organizations (71%) received drug industry support but faced issues like unpredictable funding and threats to independence. Drug firms cited unclear cooperation rules, viewing support as necessary for marketing. | Industry | COI | N |
| Hicks, (2016) | UK | Survey | Patient advocacy groups | To evaluate PAGs' understanding of compliance challenges in partnering with pharmaceutical companies. | The biggest challenges identified were transparency, project rationale, and ownership. | Industry | R&D | N |
| Hughes, (2013) | Canada | Case-study | Patient interest groups | To examine ethical issues raised by financial relationships in drug reimbursement decisions using CPC as a case study | Donors influenced patient groups by funding specific activities, aligning with industry interests. Groups often prioritized access over safety and efficiency, neglecting drug pricing issues. CPC's substantial industry funding raised concerns about independence and conflicts of interest. | Payers and HTA bodies | COI, Involvement in reimbursement decisions | N |
| Kang, (2015) | Canada, France, Germany, Italy, Japan, Spain, United Kingdom, US | Descriptive analysis of secondary data | Patient advocacy organisations | To explore the proportion of industry funds supporting PAOs in the US. | Only 6 of the 10 largest pharmaceutical companies disclosed financial transactions with PAOs in the US, while all did in France, Germany, and the UK. In 2016, 74% of disclosed funding in the US went to PAOs. | Industry | COI | N |
| Khabsa, (2020) | International | Systematic literature review | Patient groups | To synthesise evidence on financial relationships between the health industry and patient/consumer representatives. | Of 14,510 citations, 23 studies were eligible. Of these, 13% addressed financial relationships in drug regulatory processes, while 87% focused on industry funding of patient and consumer organizations, with a median of 62% reporting industry funding. | Industry | COI | N |
| Kondamuri, (2019) | US | Descriptive analysis of secondary data | Patient advocacy organisations | To characterise otolaryngologic disease PAOs and their financial ties with industry. | Half of the PAOs focused on otologic diseases, with 46.9% having publicly available donor lists. Few reported total industry donations or had conflict of interest policies. | Industry | COI | N |
| Leto di Priolo, (2012) | Europe | Survey | Patient groups | To survey stakeholder opinions on challenges, relationships, and partnerships between cancer patient groups and pharmaceutical companies in Europe. | The relationship between the pharmaceutical industry and cancer patient groups in Europe is viewed as positive but unequal and lacking transparency. Cancer patient groups aid in identifying unmet needs and developing medicines, despite concerns about their competence. | Industry | COI | N |
| Lexchin, (2019) | Canada | Descriptive analysis of secondary data | Patient groups | To examine the association between Canadian patient groups' positions on products and conflicts of interest with companies. | Patient groups declared 1896 conflicts with drug companies. Their views on drug indications were consistent, showing no significant difference from CDR/pCODR recommendations, regardless of conflicts. | Industry | Involvement in reimbursement decisions | N |
| Lexchin, (2022) | Canada | Descriptive analysis of secondary data | Patient groups | To determine if IMC member companies and patient groups publicly list donation relationships on their websites. | Reporting on donations from IMC members to patient groups in Canada is inconsistent and incomplete, making it difficult to match donations and assess their impact. | Industry | COI | N |
| Lexchin, (2022) | Canada | Descriptive analysis of secondary data | Patient organisations | To investigate the transparency of Canadian patient organizations in reporting industry relationships and financial information on their websites. | 54.6% of groups received pharmaceutical donations, with many displaying company logos and links on their websites. Few groups had policies addressing these relationships. | Industry | COI | N |
| Li, (2019) | US | Descriptive analysis of secondary data | Patient advocacy organizations | To examine conflicts of interest and disclosure practices among dermatology patient advocacy organisations. | Of 24 dermatology patient advocacy organizations with revenues over $500,000, 21% had donations over $5 million. 71% reported industry donations, but none disclosed exact amounts or uses. LinkedIn data showed at least 54% had a current or former industry executive on their boards. | Industry | COI | N |
| Makowska, (2024) | Poland | Descriptive analysis of secondary data | Patient organizations | To survey patient advocates' experiences and perceptions of involvement in HTA processes and identify issues faced during submissions. | Of 16 survey recipients, 15 responded. Respondents from Canada, England, Scotland, Wales, The Netherlands, Australia, and Taiwan reported HTA involvement, mainly at the appraisal stage. They participated in committees or provided submissions. Some respondents were unclear on their involvement's purpose, while HTA committee members better understood their impact. Feedback to patient groups was often lacking. | Industry | COI | N |
| Mandeville, (2019) | UK | Descriptive analysis of secondary data | Patient organizations | To investigate the prevalence of financial interests among patient organisations contributing to HTA at NICE. | 72% of patient organisations accepted funding from manufacturers or competitors during the appraisal year. NICE committees knew of less than a quarter of these interests due to disclosure policy gaps. | Payers and HTA bodies | COI, Involvement in reimbursement decisions | N |
| Mankell, (2021) | Sweden | Case-study | Patient organizations | To understand the nature of patient organisation representation in healthcare systems. | Patient organisations function well in representation but face democratic challenges, balancing democratic ideals with managerial practices. | Regulators, public health agencies | Involvement in regulatory decision-making process | N |
| Marvis, (2012) | Europe | Survey | Patient organizations | To describe the actions and achievements of the European rare disease patients' community. | 37% of organisations funded research, focusing on basic research, therapeutics, and diagnosis, while also providing non-financial support. | Regulators, public health agencies | Involvement in regulatory decision-making process | Y |
| Matos, (2019) | Europe | Survey | Patient organizations | To understand the role of European patient organisations in optimizing pharmacovigilance. | Out of 1898 invited patient organisations, 337 responded from 31 countries. Many want to raise awareness about ADRs (43%), but 38% lack pharmacovigilance goals. Barriers include low budget (45%), lack of resources (44%), and insufficient support from authorities (33%). More than one-third had no involvement in pharmacovigilance. | Regulators, public health agencies | RWE | N |
| McBride Folkers, (2019) | US | Descriptive analysis of secondary data | Patient advocacy organizations | To evaluate the availability of information on investigational treatments on PAO websites. | Most PAOs provided links to clinical trial information, with some mentioning the FDA's expanded access program. Information quality and presentation varied widely. | Healthcare professionals | Medical care | N |
| McCoy, (2017) | US | Descriptive analysis of secondary data | Patient advocacy organizations | To assess disclosure practices and conflicts of interest among patient-advocacy organizations. | Among 104 patient-advocacy organizations, 37% focused on cancer, 88% published donor lists, and 83% reported industry financial support. Only 1 organization explicitly rejected industry support. 36% percent had current or former industry executives on their boards, and 27% published conflict of interest policies | Industry | COI | N |
| Mercer, (2020) | Canada | Interviews | Patient groups | To examine patient group participation in the pCODR process and the challenges faced. | Patient groups value making submissions to the pCODR process but face resource challenges and uncertainty about their impact, seeking more engagement avenues. | Payers and HTA bodies | Involvement in reimbursement decisions | N |
| Minh, (2024) | Finland | Interviews | Patient organizations | To analyse the ties between the Dutch government and patient organisations. | Government significantly influences the structure, activities, and ideology of patient organisations through financial means. | Payers, HTA | Involvement in reimbursement decisions | N |
| Moreira, (2015) | Europe | Case-study | Patient organizations | To understand how patient organisations get involved in health technology assessment using the Alzheimer’s Society as a case study. | The Alzheimer’s Society evolved through stages focusing on volunteer knowledge, integrating clinical and scientific knowledge, and deepening its network to enhance evidence production. | Payers and HTA bodies | Involvement in reimbursement decisions | N |
| Mulinari, (2020) | Sweden | Descriptive analysis of secondary data | Patient organizations | To contribute to knowledge on patient organisation-industry relations in Sweden and assess commercial motives behind funding. | Forty-six companies paid 6.4m to 77 patient organisations, with few companies dominating funding linked to their drug portfolios, contributing to inequalities in resources and influence. | Industry | COI | N |
| Mulinari, (2022) | Denmark | Descriptive analysis of secondary data | Patient organizations | To analyse the pattern of industry funding of patient groups in Denmark. | Fifty-one companies paid 8.8m to 84 patient organisations in Denmark, with most funding going to high-profile diseases, creating inequalities. One company, Abbvie, dominated the funding landscape. | Industry | COI | N |
| Nahuis and Boon, (2011) | Netherlands | Case-study | Patient groups | To develop a framework explaining patient advocacy impact using social movement theory. | Patient organisations, though rarely dominant, influenced stakeholders through shared agendas and data. Their impact on reimbursement procedures was limited by a persistent market agenda for cost control. | Governments | Involvement in reimbursement decisions | N |
| Nguyen, (2022) | North America, Europe, Australia | Literature review | Patient organizations | To review the role of RDPOs in therapeutic development, focusing on their interactions across various domains such as grant funding, financial support from pharmaceutical companies, study recruitment, patient care, patient-reported outcomes, and research priorities. | Ethical and financial challenges in patient advocacy are significant. Biomedical venture philanthropy helps RDPOs with research and training, but faces sustainability issues. Advocate-industry collaborations can be source of biases. RDPOs play key roles in project promotion, recruitment, biobank creation, and registries. | Industry | R&D | Y |
| Noordman, (2010) | Europe | Survey | Patient organizations | To explore the role of patient organisations in the reimbursement of medicines in Western Europe, focusing on their cooperation with the pharmaceutical industry. | Representatives from 21 patient organisations across eight countries reported experiences; 11 small organisations did not engage in reimbursement processes or cooperate with the pharmaceutical industry. | Payers and HTA bodies | Involvement in reimbursement decisions | N |
| Obici, (2023) | International | Delphi panel | Patient advocacy groups | To develop recommendations for patient-centered multidisciplinary care for hereditary transthyretin-mediated amyloidosis | 75% consensus was reached for all but one recommendation. HCPs and PAGs agreed on core themes, but HCPs had higher consensus on several key recommendations. The highest agreement among PAGs was for nutritionist referrals, patient education, and information for families. The highest agreement among HCPs was for informed treatment decisions, genetic counseling, and monitoring asymptomatic patients. | Healthcare professionals | Medical care | Y |
| Ozieranski, (2019) | UK | Descriptive analysis of secondary data | Patient organizations | To examine industry funding of UK patient organizations. | From 2012-2016, the drug industry donated over £57m to UK patient organizations, prioritizing high-profile conditions. Transparency in payment disclosures was limited. | Industry | COI | N |
| Ozieranski, (2020) | UK | Descriptive analysis of secondary data | Patient organizations | To examine the under-reporting of payments to UK patient organisations by comparing company disclosures with annual accounts from 2012 to 2016. | Donors and recipients under-reported payments, with significant discrepancies between reported figures, indicating transparency issues in financial disclosures. | Industry | COI | N |
| Ozieranski, (2022) | UK | Descriptive analysis of secondary data | Patient organizations | To explore the financial dependence of UK patient organisations on industry funding and factors influencing this dependence. | Among 47 PAOs, 89% had a COI policy, addressing individual or institutional COIs. Only 38% restricted corporate partnerships. Most policies lacked clarity on financial disclosure and ongoing partnership review. | Industry | COI | N |
| Parker, (2019) | Australia | Interviews | Patient groups | To understand patient group interactions with the pharmaceutical industry and explore attitudes toward industry sponsorship | Patient groups had varied relationships with the pharmaceutical industry, from close partnerships to incompatible missions. Industry funding involved exchanges of money, information, and support for marketing and lobbying efforts. | Industry | COI | N |
| Pashley, (2022) | Sweden, Denmark, Finland, Norway | Descriptive analysis of secondary data | Patient organizations | To illustrate the state of transparency of disclosure of pharmaceutical industry funding of patient organisations in Nordic Countries. | Transparency issues vary between countries; Norway and Finland have less accessible and incomplete data compared to Sweden and Denmark. | Industry | COI | N |
| Patterson, (2023) | US | Survey | Patient advocacy groups | To provide an overview of rare disease PAG activities, challenges, and lessons learned in the context of collaborative R&D | Research engagement was a key goal for most PAGs (81%), especially those for ultra-rare diseases and high-budget PAGs. Overall, 79% engaged in research activities. Ultra-rare PAGs were less likely to have ongoing clinical trials. Barriers include limited funding and lack of disease awareness | Industry | R&D | Y |
| Perry, (2021) | US | Interviews | Patient groups | To describe the Clinical Trials Transformation Initiative process and tool for guiding priority patient group engagement decisions | Engagement activities improve clinical trials by enhancing recruitment, reducing costs, and meeting regulatory expectations, requiring dedicated staff, expertise, and financial resources | Industry | R&D | N |
| Polich, (2012) | International | Literature review | Patient organizations | To develop a framework for patient groups transforming experiential information into biomedical research and to identify strengths and weaknesses of patient-driven research | Rare disease patient groups contribute by conducting efficient research, compiling treatment information, and investigating novel treatments, including off-label drugs and natural products. | Industry, Other | R&D | Y |
| Rickard, (2019) | UK | Descriptive analysis of secondary data | Patient organizations | To evaluate the compliance of pharmaceutical companies with the EFPIA Code regarding payment disclosures in the UK from 2012 to 2016. | Many companies lack disclosure reports, and available reports often inadequately describe payments, lacking clarity on type, purpose, and VAT status. | Industry | COI | N |
| Rose, (2013) | US | Literature review | Patient advocacy organizations | To characterise issues undermining trust in PAOs and recommend approaches to improve conflict of interest disclosures. | PAOs often accept pharmaceutical funding, which can advance goals but also create conflicts of interest, potentially biasing actions towards donor interests. Recommendations include limiting pharmaceutical funding to a small portion of budgets, separating fundraising and policymaking roles, establishing COI review committees, and enhancing transparency through full disclosure of financial relationships. | Industry | COI | N |
| Rose, (2015) | International | Case-study | Patient advocacy organizations | To highlight collaborative efforts between GSK and Genoa Pharmaceuticals with PAOs and outline future steps to advance such collaborations. | The PFF facilitates therapy development for pulmonary fibrosis through partnerships, research networks, an advisory council, effective communication, and patient involvement in clinical trials. | Industry | R&D | Y |
| Rose, (2017) | US | Survey | Patient advocacy organizations | To describe the nature of industry funding and partnerships between PAOs and for-profit companies in the US. | Of 439 surveys, 289 were returned. PAOs varied in size and funding, with 67.3% receiving industry funding. COIs were relevant to 81.8%, and 55% rated their COI policies highly. Few felt pressured to conform to donor interests. | Industry | COI | N |
| Rozmovits, (2018) | Canada | Interviews | Patient advocacy groups | To explore reviewer and payer perceptions of meaningful patient engagement in the pCODR process. | Patient advocacy group submissions were meaningful when providing unique, relevant information. Emotional appeals and lack of transparency detracted from the credibility and effectiveness of their contributions. | Payers and HTA bodies | Involvement in reimbursement decisions | N |
| Scott, (2017) | International | Survey | Patient organizations | To survey patient advocates' experiences and perceptions of involvement in HTA processes and identify issues faced during submissions. | Of 16 survey recipients, 15 responded. Respondents from Canada, England, Scotland, Wales, The Netherlands, Australia, and Taiwan reported HTA involvement, mainly at the appraisal stage. They participated in committees or provided submissions. Some respondents were unclear on their involvement's purpose, while HTA committee members better understood their impact. Feedback to patient groups was often lacking. | Payers and HTA bodies | Involvement in reimbursement decisions | N |
| Somers, (2024) | Netherlands | Descriptive analysis of secondary data | Patient advocacy organizations | To survey patient advocates' experiences and perceptions of involvement in HTA processes and identify issues faced during submissions. | Of 16 survey recipients, 15 responded. Respondents from Canada, England, Scotland, Wales, The Netherlands, Australia, and Taiwan reported HTA involvement, mainly at the appraisal stage. They participated in committees or provided submissions. Some respondents were unclear on their involvement's purpose, while HTA committee members better understood their impact. Feedback to patient groups was often lacking. | Industry | COI | N |
| Stein, (2018) | International | Case-study | Patient advocacy organizations | To develop guidelines for effective collaborations between patient advocacy organisations and the biopharmaceutical industry. | Guidelines address patient advocacy organisations' interactions with biopharmaceutical companies, covering engagement, privacy, financial contributions, and clinical trial communication, with ethical recommendations for leaders. | Industry | R&D | Y |
| Van de Bovenkamp, (2011) | Netherlands | Document analysis | Patient organizations | To analyse the ties between the Dutch government and patient organisations. | Government significantly influences the structure, activities, and ideology of patient organisations through financial means. | Governments | Funding | N |

Abbreviations: ADR, Adverse drug reactions; CDR, Common Drug Review; COIs, Conflicts of interest; CPC, Coalition Priorité Cancer; EFPIA, European Federation of Pharmaceutical Industries and Associations; FDA, Food and Drug Administration; GSK, GlaxoSmithKline; HAS, Haute Autorité de Santé (French National Authority for Health); HCPs, Healthcare professionals; HTA, Health Technology Assessment; IMC, Innovative Medicines Canada; NCD, Non-communicable disease; NICE, National Institute for Health and Care Excellence; PAOs, Patient advocacy organizations; PAGs, Patient advocacy groups; PCGs, Patient and consumer groups; pCODR, Pan-Canadian Oncology Drug Review; PFF, Pulmonary Fibrosis Foundation; PPMD, Parent Project Muscular Dystrophy; RDPOs, Rare disease patient organizations; R&D, Research and development; RWE, Real world evidence; US, United States.

*A study was classified as focusing on rare diseases if this was explicitly stated in the text; prevalence thresholds and geographical variations in the definition of rarity were not considered in this analysis.
